# Quantitizing findings from qualitative studies for integration in mixed methods reviewing

**DOI:** 10.1002/jrsm.1403

**Published:** 2020-03-15

**Authors:** Leonie van Grootel, Lakshmi Balachandran Nair, Irene Klugkist, Floryt van Wesel

**Affiliations:** ^1^ Department of Methodology and Statistics School of Social and Behavioral Sciences, Tilburg University Tilburg Netherlands; ^2^ Department of Methodology and Statistics Faculty of Social and Behavioral Sciences, Utrecht University Tilburg Netherlands

**Keywords:** mixed methods reviewing, quantitizing, systematic review methodology, vague quantifiers

## Abstract

In mixed methods reviewing, data from quantitative and qualitative studies are combined at the review level. One possible way to combine findings of quantitative and qualitative studies is to quantitize qualitative findings prior to their incorporation in a quantitative review. There are only a few examples of the quantification of qualitative findings within this context. This study adds to current research on mixed methods review methodology by reporting the pilot implementation of a new four‐step quantitizing approach. We report how we extract and quantitize the strength of relationships found in qualitative studies by assigning correlations to vague quantifiers in text fragments. This article describes (a) how the analysis is prepared; (b) how vague quantifiers in text fragments are organized and transformed to numerical values; (c) how qualitative studies as a whole are assigned effect sizes; and (d) how the overall mean effects size and variance can be calculated. The pilot implementation shows how findings from 26 primary qualitative studies are transformed into mean effect sizes and corresponding variances.

## 1 INTRODUCTION

Recently, there has been a wide interest in enhancing synthesis methods that are suited for policy and practice—in particular, synthesis methods in which both qualitative and quantitative studies are incorporated.^1^ A mixed methods review contains both quantitative and qualitative studies, and has a major advantage over the synthesis of solely quantitative or qualitative studies in that it can lead to a very diverse understanding of a topic.^2^, ^3^, ^4^, ^5^, ^6^, ^7^, ^8^, ^9^, ^10^ However, mixed methods reviewing has not yet reached its full potential for policy and practice. More specifically, the possible contribution of qualitative studies in mixed methods reviewing requires further investigation.^1^ New ways of demonstrating the use of qualitative data in systematic reviews are welcome.

So far, when qualitative studies are used in mixed methods reviews, their role often differs from the role of quantitative studies. Qualitative studies can either function as a precursor to the quantitative work, explain quantitative findings, provide recommendations for interventions, or provide additional data for synthesis.^11^ In most cases, qualitative studies contribute to knowledge about the possible existence of relationships in a synthesis. In the matrix‐approach, for example, the qualitative evidence synthesis is used to list recommendations for interventions that hypothesize which component included in interventions could be more effective.^12^

Qualitative studies are often used to merely support quantitative studies, but recent writings have proposed a broader use of qualitative studies in mixed methods reviews. Petticrew^1^ suggests that qualitative studies might also be able to identify the range and nature of impacts and give “some sense” about the frequency of the occurrence. Moreover, qualitative studies might even provide sufficient evidence to conclude that an intervention has caused a particular outcome. This assumption, that qualitative studies can contain evidence of the nature of impacts, is supported by several scholars arguing that qualitative studies have the potential to show causal description of mechanisms, and possibly even causal explanation (eg, Reference 13, 14, 15, 16, 17). However, actually measuring the size of the impacts, or the strength of relationships, would currently still require quantitative data, according to Petticrew.^1^ This study explores new possible ways of using qualitative data in systematic reviews, in addition to only providing evidence for the *direction* of relationships to a mixed methods review. We explore the capacities of qualitative studies to estimate the *strength* of relationships and their corresponding measure of variability by reporting the findings of a pilot implementation of our quantitizing approach.

The topic quantification of qualitative data is highly debated among methodologists. This debate takes place within the broader discussion about what distinguishes qualitative and quantitative research, as the use of numerical values is often used to define quantitative research. Strauss and Corbin claim that “statistics or other froms of quantitification should not, and are not, used in qualitative research.”^18^ The primary reason to reject the use of numerical data in qualitative research is that they are incompatible with a constructivist philosophy in research.^19^ However, it is argued by many other scholars that merely the fact of having collected numerical data is not a sufficient, and a far too simplistic criterium to distinguish qualitative and quantitative methods.^19^, ^20^, ^21^, ^22^ Qualitative research uses, though it is often in an indirect way, also numerical information. Any phenomonenon “has some degree of muchness”^23^ and qualitative studies often report quantitative claims in verbal form, referred to as “quasi statistics.”^20^ Also, numbers are used in qualitative research implicitly to enable patterns in the data to emerge with greater clarity.^24^ Following this line of thinking, the transformation of textual information into numerical data is no more than providing textual data with more specification by assigning a numerical value to it.^25^ The transformation of verbal counts in qualitative studies to numbers has been attempted in the context of a systematic review.^26^

In the context of mixed methods reviewing, few examples exist in which findings from qualitative studies are used to measure the strength of relationships.^27^, ^28^, ^29^, ^30^, ^31^ Interestingly, all the example studies use different methods to transform findings from the qualitative studies into numerical information about the strength of relationships; indicating a lack of standard methods for quantification of qualitative results. Sandelowski et al^28^ calculated effect sizes from the qualitative studies by counting what proportion of the studies supported a particular finding. Voils et al^29^ counted the number of participants in all primary quantitative and qualitative studies associated with a particular factor influencing an outcome. After that, they established the prior likelihood for a participant reporting a relation, referred to by Crandell et al^30^ as the “quantitizing approach,” Crandell et al^31^ applied the “qualitizing approach” which entails the categorization of each qualitative primary study as a whole instead of each participant from each study. In this approach, all qualitative studies are labeled as validating the relationship, contradicting the relationship, or being neutral. Roberts et al,^27^ who applied the “qualitative‐as‐prior approach,” used a panel of experts to rank the barriers and facilitators found in the qualitative studies according to their importance to construct a prior distribution of probabilities for factors influencing the outcome in a Bayesian meta‐analysis. And finally, Crandell et al^30^ also used this same approach but counted the qualitative studies supporting a relationship in order to come up with a prior odds ratio based on the qualitative data.

The quantification of qualitative findings consequently requires rethinking the conceptualization and operationalization of the effect size. From a purely meta‐analytic perspective, which is adopted in the quantitizing approach from Voils et al,^29^ we consider the effect size of a study to represent the empirically measured relation between the variables for participants. The unit of analysis of the primary studies is then the participant, and the findings for every participant in either a quantitative or qualitative study are equally treated. From a qualitative research and qualitative evidence synthesis perspective, however, findings from qualitative studies are considered to be “the databased and integrated discoveries, judgments, or pronouncements researchers have offered about the events or experiences under investigation.”^32^ In this case, the unit of analysis is the study or every written finding in the study, following this line of thinking, in which the researcher has an important role in establishing the findings, we move further from the typical meta‐analytic approach and closer to the use of so‐called expert judgment in systematic reviews.^33^ Findings in the primary study are in turn the result of the researchers' interpretation of the collected data. Although the use of qualitative findings for incorporation in the context of a mixed methods review involves the findings of primary studies, the conceptualization and operationalization of “effect size” of a primary qualitative study are not necessarily equal to that of effect size in meta‐analysis. Having this said this, we emphasize that we are not devaluating qualitative research findings, on the contrary—we merely state that they should not necessarily be treated as meta‐analytic data. We choose to operationalize the effect size as the interpretation of the author more closely fits the qualitative research paradigm, and still has the advantage over using expert judgment for systematic reviews^33^ that the expert judgment is based on empirical research.

One limitation that could be overcome using this approach, is that it provides a means to come up with a measure of variability for the qualitative data set. The current quantitizing examples^27^, ^28^, ^29^, ^30^, ^31^ calculate the average mean strength of a relationship of the qualitative data but lack information about the variance corresponding to this mean. Because the variance of the mean is often used to inform about precision, for example in a Bayesian meta‐analysis using an informative prior, this information can be crucial. The only example, in which precision is mentioned, is Crandell et al^30^ who used an informative prior distribution in a Bayesian meta‐analysis based on the qualitative studies and chose an arbitrary “fairly informative” value for the precision. However, the utilization value of quantitized findings from qualitative studies could probably be enhanced if we were to determine the precision of the mean of the findings based on the variability in the actual qualitative data. The current study describes the pilot implementation of an approach for the quantification of findings from qualitative studies in which a measure of central tendency and its variance is calculated, and aims to open up discussion and debate about quantitizing in mixed methods research. The following research question is being studied: How can qualitative studies, in the setting of a mixed methods review, generate an estimate of the strength of a relationship and a variance of this estimate?

This article first presents the example with which we will illustrate our approach. We then discuss the coding procedure that was used to extract data from the qualitative studies and we elaborate on the quantification of the extracted text fragments. Finally, effect sizes and variances are calculated and sensitivity analyses are conducted to explore the rigor of the method.

## 2 EXAMPLE

One of the contributions qualitative studies can make to quantitative information in healthcare is that they can shed light on barriers and facilitators for health improvement experienced by patients.^34^ The method proposed in this study will be illustrated using the qualitative evidence synthesis of Flemming, McCaughan, Angus, and Graham^35^ concerning barriers and facilitators to smoking cessation experienced by women during pregnancy and following childbirth. Flemming et al^35^ synthesized 38 qualitative and mixed‐method studies in 41 articles following the principles of meta‐ethnography^36^ and identified four themes from which three acted as barriers or facilitators to smoking cessation during pregnancy and following childbirth. Their results show that *psychological well‐being*, *relationships with significant others*, and *perceived risks of smoking* were recurrent themes in the primary studies which either support or hinder women to quit smoking during their pregnancy or to relapse into smoking after childbirth. The qualitative evidence synthesis of Flemming et al^35^ describes *psychological well‐being*, *relationship with significant others*, and *perceptions of risk* as variables having a direct connection to an outcome, which makes these three factors eligible for inclusion in our method. The fourth theme described in the qualitative evidence synthesis, *changing connection with the baby throughout and after pregnancy*, did not constitute a direct relationship with the outcome variable smoking cessation, and was therefore not appropriate for inclusion in our approach. As mentioned in the introduction, this approach aims to provide a measure of strength for a particular relationship. Therefore, this approach is limited to research questions that evaluate a bivariate relationship. Themes were only included when they were directly related to the outcome measure. A relationship is only possible for evaluation with this approach when the findings of the qualitative evidence synthesis clearly indicate that the theme is a cause for the outcome in any way. Therefore, descriptive outcomes of qualitative evidence synthesis, which do not infer any relationship, are not included in this approach.

## 3 METHODOLOGICAL APPROACH

We present the pilot implementation of a four‐step approach to translating findings from qualitative studies into an overall effect size and its variance for the qualitative data set. The first step describes the preparation of the data set and the formulation of relationships to be measured, and the second step focuses on the organization and ranking of findings from qualitative studies using a coding procedure. Steps 1 and 2 refer to two distinct actions, but in some cases, these actions are carried out simultaneously. Step 3 describes how the effect sizes per study were calculated during the process, and step 4 describes how the overall mean and variance are calculated. A coding manual was constructed to guide the coding process. The coding manual was developed during the research process by the research team through identification and coding of studies independently, and subsequent discussion of differences until agreement. We will refer to steps 1 and 2 with the necessary examples written down in the coding manual [Supporting Information]. These steps are summarized in Table 1 and illustrated by the example concerning smoking during pregnancy.

**Table 1:** Overview of the research procedure

Step	Action	Output	Example 1	Example 2	Level^a^
1	Preparing data set and analysis	A specific relationship to be quantified, inclusion criteria for text fragments	Effect of psychological well‐being on smoking cessation	Effect of relationship with significant other on smoking cessation	Review
2	Organizing and ranking quantifiers	Organized data set holding correlations for all coded text fragments per study per relationship	“*Several women mentioned stress as a barrier for smoking cessation”* ➔ Sample quantifier, small effect, correlation = .19	*“When discussing temptations to smoke from their families*, *many women emphasized the important role their partners or spouses played*.*”* ➔ Relationship quantifier, medium effect, correlation = .23	Fragment
3	Calculating median correlation on study‐level	Correlations per study	Correlation for one study = .23	Correlation for one study = .20	Primary study
5	Calculating statistics on review level	Overall correlations and variances per relationship	Correlation = .23 and variance = .003	Correlation = .21 and variance = .002	Review

a This column describes the level of analysis for each step. Steps on review‐level concern analyses overall studies, steps on primary study‐level concern analysis overall fragments within one study, and steps on fragment‐level concern analysis of one fragment within a primary study.

### 3.1 Step 1: Preparing data set and analysis

In the first step of the approach, we defined the limits of our data set and the focus for the analysis. We chose to focus our research on smoking during pregnancy, excluding the studies dealing with relapse after pregnancy. From the original 41 papers, we analyzed 28 of the primary studies from the qualitative evidence synthesis.^35^ Seven studies were excluded because they focused on relapse after birth instead of smoking during pregnancy. Three studies were excluded because they did not focus on the relationships of interest. One study was excluded because another study, based on the same data, was also reported in another included article. If we were to treat this article as a different case, the data from this study would be used twice in the analysis whereas they were collected only once. Two dissertations were excluded because our method was specifically designed for the analysis of journal articles. The coding manual describes specific guidelines that could not be applied to a study report that has the format of a dissertation. For example, to avoid the coding repetition of findings by comparing sentences from the abstract and findings section, we require a scientific article. The quantifier and the words used for the relationship should be exactly the same in the abstract and findings section. This is further specified in the coding manual.

[Supporting Information] Subsequently, we determined the relationships to be measured. As mentioned in the introduction, we operationalize the relationships as the reported interpretation of the author for the relationship. The qualitative evidence synthesis suggested three relationships, but it should be noted that not all studies might contain text fragments in which the author indicates a relationship. We are not assuming those studies to indicate no effect (null‐effects) in the relationship, but just to contain no information about the relationship and so we leave them out of the analysis. For each included study, we isolated text fragments containing a finding. Text fragments from the studies that describe these relationships are extracted and included in the analysis if certain elements are present in the fragments. A fragment was included based on the following criteria, as described in the coding manual: (a) It should be in the abstract, findings or discussion section of the article, (b) the dependent variable smoking cessation is covered, (3) the independent variables is covered, (d) the relationship is mentioned, and (e) vague quantifier is covered. An example of how we decided to include a text fragment in the analysis is as follows:

“*Many women **perceived*** ***quitting during pregnancy*** ***as too stressful** for the fetus*, *potentially causing harm or even miscarriage*.”^37^ ^i^

The elements in this text fragment that are essential for inclusion are the reference to quitting smoking, and the reference to the perception of risks, and the relationship between those two elements. We present more examples of how we included text fragments in the description of step 2, because steps 1 and 2 are in some cases carried out simultaneously. A more extensive overview of these definitions and the conditions for inclusion in step 1 can be found in the coding manual (available as online material).

### 3.2 Step 2: Organizing and ranking quantifiers

The coding manual describes two types of information for which text fragments are coded. [Supporting Information] Before starting the development of the actual coding manual, (LvG) had studied six of the included primary studies for information and identified “vague quantifiers” with regard to two types of information that seemed to be present in text fragments while reading the studies: (a) information with regard to the sample and (b) information with regard to the relationship itself. Vague quantifiers are present when researchers imply numbers using quantitative designations which refer to either participant like “many women,” “the majority of the women,” and “some women” or to the relationship like “is a strong coded text fragment for,” “has a large effect on,” or “is a recurrent theme.”^28^, ^29^, ^30^ By obtaining counts, qualitative researchers can quantitize vague quantifiers.^38^

(LvG) discussed the findings with (FvW), and consequently (FvW) also studied six studies independently. Based on the findings with regard to the vague quantifiers, (LvG) and (FvW) coded the abstract, results, and discussion sections of the studies as most studies followed an amended‐experimental‐style.^39^ Next (LvG) and (FvW) again discussed their findings which resulted in the first version of the coding manual. (LvG) used the first version of the coding manual to code all remaining studies in the software NVivo 11.^40^ For reliability purposes, eight of these studies were also coded by a third coder (LBN) independently. The coding manual was an ongoing topic of discussion in the research team and not considered finished until full consensus was reached. It resulted in an extensive description of the inclusion criteria of text fragments from the primary studies containing qualitative findings and the categorization of these text fragments into the two categories. In addition, a complete list of all vague quantifiers identified can be found in the coding manual.

The first type of quantifiers, *Sample quantifiers* (SQ), is found in fragments in which the authors refer to the relationship of interest using vague quantifiers with regard to the sample. As the coding manual specifies, a fragment was categorized as SQ, when the author implies numbers using quantitative designations referring to participants. The following fragment, holding a statement about the relationship between psychological well‐being and smoking cessation, illustrates how the coding manual was applied to text fragments concerning SQ:

*“**Most** respondents felt that pregnant women would **give up cigarettes** if they could*, *but **needed a ‘crutch’ for life hardship**.”* 41

The elements that are essential for inclusion of the fragment in our analysis are the presence of a vague quantifier, and the relationship of interest (both boldfaced in the example). In this case, all the necessary elements for coding were captured in one sentence, which makes this fragment a meaningful whole. The elements “needed a ‘crutch’ for life hardship” and “give up cigarettes” classify this fragment as the relation between *Psychological well‐being* and smoking cessation. Finally, the element “most respondents” represents the vague quantifier regarding the sample. This fragment was therefore coded as *Psychological well‐being*, *SQ*. Another example of a fragment coded in this category is:

***“Many women (9/13)** stated that they would have **given up smoking** if they had been given **proof that it was dangerous***, *that the baby would be harmed.”* 42

The second category, *Relationship Quantifier* (RQ), is used for text fragments in which the author specifies the strength of the relationship between the two variables using a vague quantifier. In this category, the vague quantifier does not relate to the sample or frequency of the occurrence, but to the description of the relation between the variables itself as interpreted by the author. Often, the two variables are linked in a sentence explaining one to be the consequence or cause of the other. As the coding manual specifies, a fragment was categorized as RQ when researchers imply numbers using quantitative designations that refer to the relationship. The following example illustrates how fragments coded as RQ were selected and coded.

*Smoking was a familiar and **necessary tool** to cope*.”^43^

In this example, the elements “smoking” and “to cope” classify this fragment as the relationship between *Relationship with significant others* and smoking cessation. Finally, the element “necessary” represents the vague quantifier regarding the relation. This fragment was therefore coded as *Relationship with significant others*, *RQ*. Another example of a fragment coded in this category:

*“In line with this suggestion*, *both women and key informants **stressed** that **smoking by partners***, ***family members and friends deterred quit or reduction attempts** and thus conveyed the need for programs and services that target partner and family smoking.”* 37

From the 28 studies, two studies did not contain any vague quantifiers concerning one of the relationships and so these dropped from the analysis. In the 26 remaining studies, we found a difference between the number of coded fragments as SQ or RQ. Fragments containing quantitative information with regard to the sample (SQ) were most present in the studies. Fragments that contain quantitative information about the relationship (RQ) were found less often. We found a total of 73 fragments concerning *Psychological well‐being*, 74 fragments concerning *Relation with significant others*, and 68 fragments concerning *Perceptions of risk*. Table 2 shows the total number of coded fragments per relationship and per type of quantifier, and the total number of sources (studies) from which the fragments were selected.

**Table 2:** Number of coded fragments and studies per relationship and category

	Psychological well‐being	Studies	Relation with significant others	Studies	Perceptions of risk	Studies	Total
SQ	38	18	42	16	53	16	133
RQ	35	12	32	17	15	8	82
Total	73		74		68		

### 3.3 Ranking and quantification

In order to provide numerical information about the strength of the relationship, the text fragments were quantitized. More specifically, for each text fragment that refers to one of the three relationships, the coder scores a vague quantifier according to how strongly it represents the relationship. A dichotomous scale is often used for quantitizing (eg, Reference 39)—present or not present, but that solely allows us to ascertain that an effect is present, and therefore would “undercut the ability to capture the nuance and subtlety of particulars in qualitative studies.”^25^ The reviewer scores the text fragments by judging the vague quantifiers on an ordinal scale. We chose an ordinal scale because this allows us to code the *extent* to which a relationship is present.

All three coders listed and ranked the vague quantifiers from the studies based on how strongly they represent one of the three relationships, and then constructed a three‐point, five‐point, and seven‐point scale from the ranking. All rankings are specified in the coding manual [Supporting Information]. For validation purposes, a blind peer also independently ranked the quantifiers. In addition, we asked another blind peer who is a native speaker to check our rankings. Both peers validated our findings. (LvG) coded the first six studies to decide which scale would best fit the data. For all six studies, a categorization of five different values to indicate the strength deemed to be sufficient. We chose to use a five‐point scale with the labels “very small” (2), “small” (3), “medium” (4), “large” (5) and “very large” (6).

Negative values could also be added to the scale (−1, −2, −3, −4, and − 5). They would be used when a text fragment is encountered which indicates an effect in the opposite direction. We did not come across these effects, but other researchers applying this approach could come across groups of studies that include contradictions and negative cases.^44^ The vague qualifier would then be used in the opposite way, for example stating the following sentence (fictional):

**“Some women** stated that new knowledge of the **risks of smoking** for the fetus actually made them less likely to successfully **quit smoking**.”

The labels were then transformed to effect sizes. We chose to use correlation coefficients as “effect sizes,” because our aim is to describe how the scores of one measure relate to the scores of another measure for that sample, indicating a cause of the factors on the effect smoking cessation. Note that we do not imply causality in the strict sense that we consider all alternatives for smoking cessation be ruled out—already given by the fact that we consider three different causes for smoking cessation—but we do assume that the factor precedes the outcome. Therefore, we do use the labels “independent” and “dependent” variables. The scores are, however, not assigned to the independent and dependent variables as their unique variances are not measured. It is merely assumed that we can estimate how strongly these variables are correlated.

In order to come up with meaningful and realistic values to be assigned to the five‐point scale for measuring the correlations, we have asked a researcher with expertise in research in healthcare interventions what an appropriate range of effect sizes is for this field. To determine a range that is firmly grounded in the literature, he made a selection of seven recent systematic reviews in public health that he, based on his knowledge of the field, deemed relevant for our case. We have collected all effect sizes from the primary studies that were reported in these seven recent systematic reviews to get an idea of the range of effect sizes that one can expect from interventions in public health. A total of 69 effect sizes were extracted. We found an average correlation *r* of .22. The correlation range was .01 to .54. We divided these data into six equal parts, and we selected the values of the effect sizes corresponding with the percentile 16.7, 33.3, 50, 66.7, and 83.3. The values corresponding to these percentiles were .13, .19, .23, .27, and .31, and these numbers were assigned the labels very small, small, medium, large, and very large respectively.

### 3.4 Step 3: Calculating statistics on the study‐level

We calculated a median correlation per relationship per study based on the correlations assigned to the text fragments. The assigned correlation scores to the fragments were not normally distributed within the studies, and the scale used for coding was measured on ordinal level, we preferred to use the median as a measure for central tendency over the mean. Every assigned correlation of a text fragment represents one coded text fragment for a relationship. The correlations assigned to a text fragment are denoted *r*_*ijk*_ for the *k*th fragment of study *i* (*i* = 1, …, 26) and relationship *j* (*j* = 1, 2, 3). Step 3, therefore, concerns the estimation of the median correlation of a specific relationship per study. Note that we have not calculated a measure of variability within studies, which would in a classical meta‐analysis, when used as weights per the study, reflect the amount of information the study contains.

The results are presented in Table 3. The number of fragments varied between 1 and 13.

**Table 3:** Median, and number of fragments for the three relationships

		Psychological well‐being			Relationship with significant others			Perception of risks		
Study	Author	*m*_*i*1_		*n*_*i*1_	*m*_*i*2_		*n*_*i*2_	*m*_*i*3_		*n*_*i*3_
1	Abrahamsson	0.23		5				0.13		3
2	Arborelius	0.27		3	0.23		1	0.19		3
3	Borland	0.27		3	0.21		2	0.23		2
4	Bottorff				0.23		7			
5	Bull	0.27		4	0.13		1	0.13		3
6	Cottrell	0.19		13	0.27		2			
7	Dunn	0.13		1	0.27		6	0.21		8
8	Edwards	0.16		2	0.23		3			
9	Haslam	0.19		3	0.19		3	0.19		3
10	Herberts							0.19		1
11	Hotham	0.23		4	0.19		3	0.25		2
12	Howard	0.23		1	0.23		1			
13	Lawson				0.23		1			
14	Lendahls							0.27		1
15	Maclaine	0.21		2	0.23		3	0.23		4
16	Naughton				0.13		1	0.23		21
17	Nguyen	0.31		1	0.19		12			
18	Nichter	0.21		4	0.19		5	0.19		3
19	Pletsch	0.31		3						
20	Quinn	0.29		2	0.27		1	0.29		2
21	Thompson				0.19		10			
22	Tod	0.23		5	0.20		4	0.13		2
23	Wakefield	0.23		3	0.27		2			
24	Wigginton	0.13		1	0.19		1	0.27		5
25	Wood	0.27		13	0.19		4	0.21		4
26	Zieland				0.20		2			

*Note:* *m*_*ij*_= median correlation, *n*_*ij*_= number of fragments.

### 3.5 Step 4: Calculating the overall mean and variance

The median per study (*m*_*ij*_) served as input for the calculation of estimates of the correlation average coefficient (*R*_*j*_) and its variance (*S*^2^_*j*_). Table 4 shows the results. The medians per study approximated a normal distribution which allowed for an overall mean correlation and corresponding variance to be calculated.

**Table 4:** *Mean effect sizes*

	*N*_*j*_	*R*_*j*_	$S_{j}^{2}$
Psychological well‐being	19	.23	0.003
Relation with significant others	21	.21	0.002
Perceptions of risk	17	.21	0.002

*Note: N*_*j*_ = number of studies per relationship, *R*_*j*_ = Mean correlation of the qualitative data set for predictor *j*, $S_{j}^{2}$ = variance of the qualitative data set for predictor *j*.

### 3.6 Sensitivity analyses

During the pilot implementation, we have made certain assumptions. Firstly, for the quantification, we chose a five‐point scale. In order to check the influence of these two choices on the results, we have performed a sensitivity analysis. Secondly, we noticed that some of our included studies only hold one text fragment indicating a relationship, which we decided to include in the calculations for they make a contribution to the overall estimated relationship. However, one could argue that these measures are unreliable, as the measured correlation in the tet fragment cannot be corroborated by a second text fragment in the study. Therefore, we performed a sensitivity analysis excluding the one‐indicator studies to examine whether their inclusion has any influence on the overall mean correlation and variance.

The quantification of fragments was based on the assumption that a five‐point scale for the strength of correlations would fit the data best. In order to check whether the results are sensitive to this assumption, we recalculated the values using the seven‐point scale and three‐point scale. The correlations corresponding to the seven‐point scale were .09, .15, .20, .23, .26, .29, and .33. The correlations corresponding to the three‐point scale were .15, .23, and .29. Table 5 shows that there are small differences in the mean correlations when the scaling changes in these ways. Although no clear pattern is evident from the results of the sensitivity analyses, the results do show that for relationships with significant others and perceptions of risks, the mean correlation is somewhat lower. For psychological well‐being, there is no difference. The variances vary slightly. These analyses show that the results are marginally sensitive for the range of the scale for coding.

**Table 5:** *Results sensitivity analysis for different scaling of effect size values*

	Scaling	*R*_*j*_	$S_{j}^{2}$
Psychological well‐being	Seven‐point	.23	0.001
	Five‐point	.23	0.003
	Three‐point	.23	0.001
Relationship with significant others	Seven‐point	.18	0.001
	Five‐point	.21	0.002
	Three‐point	.20	0.002
Perceptions of risk	Seven‐point	.18	0.003
	Five‐point	.21	0.002
	Three‐point	.21	0.002

*Note: R*_*j*_ *=* mean correlation of the qualitative data set, $S_{j}^{2}$= variance of the qualitative data set for predictor *j*.

The sensitivity analyses Table 6 show that there are only very small differences in the mean correlations and variances when one indicator studies are excluded. We, therefore, believe that in this data set, the inclusion of one‐indicator studies is not problematic.

**Table 6:** Results sensitivity analysis for exclusion studies with one indicator

		*N*_*j*_	*R*_*j*_	$S_{j}^{2}$
Psychological well‐being	Inclusion	19	.23	0.003
	Exclusion	15	.24	0.002
Relationship with significant others	Inclusion	21	.21	0.002
	Exclusion	15	.22	0.001
Perceptions of risk	Inclusion	17	.21	0.002
	Exclusion	14	.20	0.003

*Note: N*_*j*_ *=* number of studies per relationship, *R*_*j*_ *=* mean correlation of the qualitative data set, $S_{j}^{2}$= variance of the qualitative data set.

## 4 DISCUSSION

This study reported the pilot implementation of the quantitizing approach. We discuss some insights and limitations that the pilot implementation provided with regard to the coding, operationalization of the effect sizes, sampling, modeling, and publication bias. Finally, we emphasize the additional value of our method for mixed methods reviewing.

### 4.1 Coding

The coding manual developed in the study proved to be very useful. In particular, the distinction between fragments as either SQ or RQ assisted in the identification and structuring of the findings in the studies. These categories provided a framework to analyze the studies for the extraction of different types of findings that indicate the strength of the relationships. The distinction is evident and frequently present in the studies. In addition, this distinction is in line with Sandelowski^45^ who discussed vague quantifiers in qualitative studies that either refer to “participants” or to “themes.” We emphasize that the potential of the coding manual developed in this study should be further explored in future research applying our method.

Although the coding manual was helpful, coders found the experience of coding for vague quantifiers to be complex. We emphasize the need for trained coders who are familiar with the diversity of reporting styles in qualitative studies. More specifically, unraveling causal mechanisms from fragments proved difficult due to ambiguity in the sequence and cause of effects. Especially when the studies were less clear in the statement and the wording of findings, it was challenging to identify and classify the nature of the relationship in a fragment. In these instances, discussion between coders was necessary to determine whether a fragment should be included or not. This experience led us to clarify the description of the inclusion criteria for text fragments repeatedly. Therefore, in future applications of this method, we recommend that the coding manual be made as detailed as possible with respect to the criteria for the inclusion of text fragments. Furthermore, we emphasize the importance of using multiple coders for the development of the coding manual and the actual coding.

The coders experienced some difficulties with the coding of studies that incorporated literature references in the results section, making it difficult to determine whether statements are actual findings from the study at hand or from other studies.^39^ Some of the fragments were considered too ambiguous with regard to their source. For that reason, these fragments were left out of the analysis. Having said this, we emphasize that this issue does not necessarily undercut the appropriateness of these types of studies for inclusion in the proposed method. It could lead to a relatively low number of coded text fragments (which means less precision) for these studies compared to other studies, but the studies could still contribute to the overall mean.

### 4.2 Operationalization

Although the distinction between PQ and SQ proved useful in coding, the operationalization of the effect sizes was not straightforward from the start. The assignment of numerical values to the effect sizes is a crucial part of the approach. We based the numerical values on the effect sizes reported in recent systematic reviews, selected based on expert judgment. One problem we encountered with this choice was the possibility that the effect sizes in the literature were not equally distributed in the extremes of the scale. For example, if the literature would have relatively few studies having effect sizes greater than .31 (maximum effect size) compared to studies having effect sizes smaller than .13, the mean effect size would be systematically upwardly biased. A different option that we considered is to use the guidelines of Cohen^46^ who uses correlations of .1, .3, and .5 for the three middle values of the scale. This option would provide an existing and often used framework for assessing the magnitude of the effect sizes. However, the reason why we have chosen to work with values grounded in literature is that the guidelines of Cohen are less realistic for public health interventions. This assumption was also supported by the literature that showed an average effect size of .22 and a range of .01 to .54. Using the guidelines of Cohen^46^ on this data set would therefore probably lead to an overestimation of the average effect size. We, therefore, emphasize that the scaling used in approach is only one way to determine the range of effect sizes appropriate for a field and that further research concerning the establishment of the appropriate range of effect sizes for the discipline under investigation is necessary. Furthermore, a combination of methods in order to increase criterium validity might also be worth investigating.

A related, but distinct issue is that the values that were found in the literature were forced into a five‐point scale, which may have influenced the correlation per study to be biased toward the center of the scale. The values that we found in the literature ranged from .01 to .54, whereas the ranges on our five‐point scale were .13 and .31; based on the five points in the distribution. In the case that the lowest value of .13 would be an underestimation of the “actual” lowest correlation or the value of .31 would be an overestimation of the “actual” highest correlation, the correlation per study would systematically biased—downwoard and upward, respectively. The sensitivity analysis using the seven‐point scale gave an indication that the results would be slightly different when the scale would rank from .09 to .33, and so the restriction of the range should be mentioned as a potential source of bias. The challenge for reviewers applying this approach face, is to weigh the desired precision with which to code the strength indicated in the text fragment against the usability in the coding process. Ten‐point scales or even larger scales could also be used for coding but could prove challenging when for example the variation in vague quantifiers is limited in the data set. The reviewer must choose between having a manageable coding system and being precise with regard to the numeric values being assigned to the labels. We recommend to use several scales and perform sensitivity analyses.

A possible extension of the approach suggested in this study would be to calculate a measure of variability for the effect size per study. For the pilot of this approach we did not calculate a measure of variability per effect size. From a meta‐analytic perspective, this would suggest that there are no within‐study variances, meaning that the observed mean correlation is the “true” mean for that relationship. However, as this is not a classical meta‐analysis, the interpretation of the variability per study would not automatically reflect the same concept, but is open for discussion. For example, it could inform the reviewer how the found correlation varies between text fragments within one study. One possibility to conceptualize this measure would be to interpret it as the consistency with which the author has reported the findings. The inverse of the variances per study could, in turn, be used as weights in the calculation of the overall effect size for the qualitative data analysis. This practice does, however, require that the distribution of the effect sizes per study allow for the calculation of study variance. In our study, the effect sizes were not distributed normally and the distances between the values on the scales were not equal, leading us to use medians instead of means. If in a future study, normality of the effect size per study could be assumed, means could be calculated accompanied by a natural variance per study. Another option would be, if we were willing to alter our conceptualization of the measure of variability, to conceptualize the RQ as the effect size and the SQ as its corresponding variance if somehow matching them would be possible. That would be even closer to conducting a classical meta‐analysis, for the unit of analysis would then change from the text fragment to the participant. Having said this, we emphasize that the absence of within‐study variances in our sample does not mean that there is no error variance in the measurement within studies, that largely depends on the reviewers' interpretation of within‐study variances. It just means that we have not found a way to operationalize the variance per study for our approach yet as our data set did not allow for the calculation of within‐study variances‐ underlining the importance of more examples applying our approach in which it may be possible to calculate those.

### 4.3 Sampling

The sample of studies is from an existing review and therefore, can include studies that do not contain indicators of the relationships. In our approach, we have only used the studies reporting information on the correlation for calculating the overall effect size. From the other studies, we do not know whether the correlations were measured ‐ we only know that the interpretations of the authors about the relations were not reported in the article. From a meta‐analytic perspective, the effect sizes for these studies could be considered to be missing values. We cannot assume that these values are missing ad random. The absence of the report on a particular relationship might also indicate a negative effect: For example, if asked “what factors make you keep smoking?” the omission of a given factor may indicate that it is not important; which would indicate a negative effect size in our approach. Consequently, this approach inevitably involves the exclusion of studies from the sample, which might lead to an overestimation of the average effect size. In future applications of this approach, reviewers could check which types of information were elicited in the primary studies and account for that in their inclusion criteria for the sample.

The sample of the example qualitative evidence synthesis that we have used contains studies carried out in a variety of traditions and methodologies in qualitative research. Our research did not focus on the differences between these traditions. Nevertheless, it might be the case that studies based on certain traditions or methodologies in qualitative research are more suitable for inclusion in our method than others. Furthermore, it might be the case that there is a difference in reporting between authors of studies that support the work of Sandelowski on counting in qualitative research^28^ and those who do not, as this method relies heavily on the assumption of vote counting. It would be interesting to further examine how widely applicable our method is with regard to differences in assumptions that qualitative studies are based on.

### 4.4 Modeling

An important limitation that we came across in developing this approach concerns the assumption of the type of model to be analyzed. We assumed that the example of smoking cessation in pregnancy contained only direct effects. This means that we considered a relatively straightforward model for the application of this method. It might be more difficult to infer indirect effects (moderation or mediation effects) from qualitative studies. Van Grootel et al^47^ developed a method to formulate indirect effects from the outcomes of theory‐building qualitative evidence syntheses. Further research, to investigate whether it is possible to infer such effects from primary qualitative studies, is indicated, opening the possibility of exploring the application of the coding manual from this study to complex interventions that contain indirect effects as analyzed within other meta‐analytic models.

### 4.5 Publication bias

An underexposed concern in any form of qualitative evidence synthesis is the topic of publication bias, that is, dealing with the problem of unpublished studies that are not included in the data collection process in a review study.^48^ When the excluded studies account for a systematic distortion of the represented population, publication bias could influence synthesis results. Toews et al^49^ explored the concept of publication bias in qualitative research and found that reviewers and editors of scientific journals are likely to reject qualitative studies that are of poor quality or of which the reporting is of poor quality. In this study, the problem of publication bias is difficult to deal with as we are constrained to the decisions made on the inclusion of studies in the earlier qualitative evidence synthesis. The problem, however, remains that studies that were excluded or not found in the qualitative evidence synthesis are also not represented in our analysis. Hence, since the approach piloted in this paper relies heavily on the emphasis of wording in reporting, publication bias could have influenced the overall estimates of the mean and precision if unpublished studies would typically have a different reporting style. However, at this moment, there is no appropriate method available to recognize and account for publication bias in qualitative evidence synthesis. Further exploration is required to determine the extent to which this approach is vulnerable to publication bias and, if so, what procedures are appropriate in order to mitigate this issue.

A second potential threat for the validity of this approach concerns truncation bias.^50^ Truncation bias occurs when the author is limited to a maximum number of words for a journal article, which could influence the extensive description of the findings in the article and in turn the number of text fragments eligible for inclusion in the piloted approach. If we were to assume that the left out text fragments (due to truncation) would systematically differ from those included, the possibility is that truncation would have influenced the measure of strength per study. Concerning the variance per the study, if we were to assume that the text fragments that are possibly left out were consistent with the text fragments holding the reported findings, it could be that we would be overestimating. Unfortunately, there currently is no method to determine whether truncation bias influenced our findings, and, if it did, how it influenced our findings. We recommend further research on the sources and consequences of truncation bias in order to take this issue into account in the application of the approach in this approach.

### 4.6 Conclusion

The approach piloted in this study reduces the possible bias in the interpretation of vague quantifiers by using multiple coded text fragments for the calculation of the effect size and an overall variance. First, we have shown how the data set and analysis can be prepared by describing the inclusion criteria for text fragments to be included in the sample per study. Second, we have explained how we operationalize the variable strength of the relationship, how we have come up with a ranking for the vague quantifiers and how we assigned numerical values to each of them. Third, we have calculated the statistics for each primary study. Finally, we have calculated the overall mean and variance of the qualitative data set. We conclude that the approach piloted in this study might be very useful in the preparation of a mixed methods review, as it provides a more reliable estimate for the strength of a relationship than existing methods do. Consequently, this approach might be appropriate to serve as input for a qualitative‐as‐prior approach in a Bayesian meta‐analysis. As this paper reports on the pilot implementation of a new approach, we invite other researchers to build on this approach and share their comments and experiences.

## CONFLICT OF INTEREST

The authors declare no potential conflict of interest.

## Supporting information


**Appendix** **S1.** Supporting Information

## Data Availability

The data sets generated during and/or analyzed during the current study are available from the corresponding author on reasonable request.

## Endnote

i
Note: In all text fragments, emphasis added
