# Supplementary material for: Quantitizing findings from qualitative studies for integration in mixed methods reviewing
Source: Res Synth Methods. 2020 Mar 15;11(3):413–25. doi: 10.1002/jrsm.1403 (PMC7317911; doi:10.1002/jrsm.1403)
Supplement: Supplementary file 1 — Appendix S1. Supporting Information [file JRSM-11-413-s001.docx]

**Coding manual corresponding to paper: Van Grootel, L., Balachandran Nair, L., Klugkist, I., & Van Wesel, F. *Quantitizing findings from qualitative studies for integration in mixed methods review***

This coding manual is developed to provide detailed information about the coding procedure in Step 1, 2 and 3 described in the corresponding paper. We describe all steps from the preparation of the analysis to the quantification of the findings. We first present an overview of the conditions for inclusion of text fragments in the analysis corresponding to Step 1 (*Preparing dataset and analysis*) in Table 1. The detailed elaboration on these conditions will follow in the text below. Thereafter, we present the detailed information corresponding to the actions in step 2 (*Organizing and ranking quantifiers*).

Table 1

*Overview of conditions for the coding procedure*

|  |  |
| --- | --- |
| Condition 1 | Limit to text fragments from abstract, findings, conclusion and discussion sections of publication |
|  |  |
| Condition 2 | Dependent variable is covered in the text fragment (smoking cessation) |
|  |  |
| Condition 3 | Independent variable is covered in the text fragment (Psychological well-being, Relationship with significant others, Perceptions of risks) |
| Condition 4 | Relationship between dependent and independent variable is mentioned in the text fragment |
| Condition 5 | Vague quantifier is mentioned in the text fragment |
| Exception 1 | Text fragments containing exact replication are excluded |
| Exception 2 | Text fragments with findings from other studies are excluded |

**Step 1: Preparing dataset and analysis**

This section provides an overview of the conditions for inclusion of text fragments in the analysis. Condition 1 concerns which part of the article should be coded; Condition 2 concerns the conceptualization of the dependent variable; Condition 3 concerns the conceptualization of the independent variables; Condition 4 indicates that a relationship between dependent and independent variable should be present in the text fragment; and Condition 5 concerns the presence of a vague quantifier. Furthermore, we describe two exceptions to the rules of inclusion. Examples are given to illustrate an action. In the provided examples, the part of the example referring to the condition or exception is written boldfaced.

*Condition 1: Part of the article*

The abstract, findings and conclusion/discussion sections of the articles should be coded. Coded text fragments can cover just one sentence or a whole paragraph and anything in between. Some of the articles in the dataset contain both quantitative and qualitative data. We limit our inclusion of fragments to those originating from the qualitative data. For more information about how qualitative findings can be extracted from mixed-method studies, we refer the reader to Boeije, Van Wesel and Slagt^1^.

*Condition 2: Dependent variable smoking cessation is covered*

The dependent variable is explicitly mentioned when smoking cessation (or quitting smoking, smoking reduction, controlling smoking) is literally stated. The dependent variable is implicitly mentioned when no direct reference is made to smoking cessation in the coded fragment, but when the relationship with smoking cessation can be interpreted based on the context. This happens when the section in which a fragment is discussed concerns smoking cessation in relationship to the independent variable, but the presence of the dependent variable is not repeated in the coded fragment itself. The following example illustrates this situation:

Beginning of the section in the paper:

*“The women in this study and previous work (Haslam et al., 1997) were aware of the risks*

*but this was not sufficient to make them* ***quit****.”^2^*

Coded fragment later in the same section:

*“Previous uncomplicated pregnancies may make some women believe they have* ***‘personal immunity’ against the risks.****”^2^*

It could also be that the study as a whole deals with the relationship between smoking cessation and the independent variable, and this is mentioned in the introduction of the study, but not in all sections of the results.

*Condition 3: Independent variable is mentioned in the text fragment*

We analyze three separate bivariate relationships. In this section, we elaborate on the definitions of the independent variables in those relationships.

3a: Psychological well-being

Psychological well-being covers stress and all other text fragments in which “feeling better/relieved due to smoking”, “self-efficacy”, “self-esteem” are mentioned. Psychological well-being can either be mentioned as a barrier for quitting (“the thought of quitting smoking makes a lot of the women feel more stressful”) or as a facilitator to keep smoking (“all participants mention feeling relieved due to smoking”). The following quotation shows an example of how psychological well-being can be phrased.

*“Smoking was a familiar and necessary* ***tool to cope.****”^3^*

In this case, the relationship has a suggested direction: higher psychological well-being leads to higher probability of successful smoking cessation. Although we did not find any text fragments in our sample suggesting otherwise, we do want to stress the possibility. A fictional example would be:

*“Many women indicated that the* ***stress*** *they experienced actually made them adamant to quit smoking.”*

3b: Relationship with significant others

Relationship with significant others covers all text fragments that refer to the influence of partners, family or friends on the cessation attempts of the pregnant women. These text fragments can either be formulated as a barrier for smoking cessation (“a lot of women mention that the smoking of their partners in front of them makes cessation more difficult”) or as a facilitator to keep smoking (“if the partner keeps smoking, some women tend to follow that example to avoid conflicts.”). The following quotation shows an example of how relationship with significant others can be phrased.

*“Bearing the guilt of smoking during pregnancy and facing the difficulties of trying to stop and stay stopped* ***while other relatives and friends continued to smoke*** *were commonly cited experiences.”^4^*

In this case, the relationship has a suggested direction: more involvement of significant others leads to higher probability of successful smoking cessation. Although we did not find any text fragments in our sample suggesting otherwise, we do want to stress the possibility. A fictional example would be:

*“The involvement of the* ***partner in the intervention*** *sometimes appeared to have the opposite effect: the extra burden of* ***dealing with a partner*** *that also attempted to quit smoking made women less successful in their cessation attempts.”*

3c: Perceptions of risks

Perceptions of risks of smoking covers all text fragments that refer to the expressed doubt that smoking during pregnancy would be hurtful in any way. This can either be a lack of knowledge, skeptical attitude towards science or physicist, belief in friends or family members that have had healthy children, or just different attitudes towards smoking and pregnancy. Text fragments referring to cognitive dissonance are also included if they are in any way related to smoking cessation. Notice that this relationship contains a double negative: when pregnant women are not aware/open of/to the fact that smoking hurts the unborn child, they will be less likely to quit smoking. Often text fragments will be formulated as a facilitator to keep smoking (“a lot of women belief smoking will not actually hurt the baby.”).

*“Many women (9/13) stated that they would have given up smoking* ***if they had been given proof that it was dangerous****, that the baby would be harmed.”^5^*

In this case, the relationship has a suggested direction: more involvement of significant others leads to higher probability of successful smoking cessation. Although we did not find any text fragments in our sample suggesting otherwise, we do want to stress the possibility. A fictional example would be:

*“Some women suggested that* ***learning more about the risks involved*** *would not have motivated them in their cessation attempts: it would only have made them more aware and therefore more uncomfortable, leading to a less successful cessation attempt.”*

*Condition 4: Relationship is mentioned*

The text fragment should not only contain the dependent and one of the three independent variables, it should also describe a relationship between those two. These relationships can be stated positively (facilitator for quitting) or negatively (barrier for quitting). There can be an explicit reference to the relationship with smoking cessation or an implicit reference. In an explicit reference, words as “smoking cessation” or “quitting smoking” are linked to an independent variable in the text fragment. An implicit reference is made when the relationship is not literally mentioned, but can be interpreted from the context of the paper. In the following example, the relationship is implicitly mentioned. This text fragment does not literally include smoking cessation, but is extracted from a paragraph describing all kinds of barriers for quitting smoking during pregnancy and can therefore be included in the analysis.

*“However, as soon as someone challenged the smoking, a defensive posture was taken, which served to facilitate living with the feeling of guilt, thereby* ***enhancing well-being****. In addition,* ***psychological well-being*** *during the pregnancy declined with time for some women and* ***smoking became even more important****.”^6^*

**Step 2: Organizing and ranking quantifiers**

This section of the coding manual describes Step 2 of the method: organizing and ranking the vague quantifiers. First, we describe the fifth condition for inclusion in the analysis; the presence of a vague quantifier in the text fragment; then we elaborate on the distinction between population and relationship quantifiers. Third, we explain how the vague quantifiers can be assigned a rank and how these ranks relate to correlations.

*Condition 5: Vague quantifier is mentioned*

The text fragment should contain a vague quantifier. As was mentioned in the paper, vague quantifiers are present when researchers imply numbers using quantitative designations which refer to either participants like “many women”, “the majority of the women”, and “some women”^7-9^ or to the relationship like “is a strong indicator for”, “has a large effect on” or “is a recurrent theme”. By obtaining counts, qualitative researchers can quantitize vague quantifiers^10^.

For inclusion of a text fragment in the analysis, the quantifier needs to refer to the relationship between the dependent and one of the three independent variables. This reference can be explicit or implicit. When the connection between the relationship and the quantifier is not explicitly mentioned but can be interpreted from the context, the text fragment should be included. An example of such a situation:

*“Before pregnancy they had all expected that they would* ***stop smoking*** *as soon as they got pregnant, because they wanted the best for their babies. Later, when the pregnancy was a fact, they were confronted with their actual capacity to stop smoking. Some of them did succeed, some did not. Among all the interviewees, and particularly among those who did not succeed, a conﬂict was generated between not giving the best to their baby and the gains of smoking. In this conﬂict, various elements of the smoking, like experiences, hearsay, scientiﬁc facts, social circumstances and well-being, were struggled with.* ***All women*** *were found to be* ***more or less familiar with facts about the risks of smoking, although at the same time some of them still trusted more in their personal experiences and hearsay of the risks.****”^6^*

In some cases, the quantifier refers to descriptive information on one of the variables but not to the relationship. The following example illustrates this situation in which the vague quantifier relates to the independent variable but not to the relationship, and therefore this fragment is therefore excluded.

*“****None of the interviewed respondents*** *reported receiving help in* ***educating their partner/family about the risks of active and passive smoking****, thus reducing the positive role they could play in smoking cessation.”^11^*

Condition 5a: Population Quantifier (PQ)

The authors states which part of the sample supports the relationship between the dependent and one of the independent variables. These statements contain (vague) quantifiers. We have listed all the identified population quantifiers in Table 1 (first column). The (vague) quantifiers in the text fragments can also refer to the uncertainty in which a statement about the population is made.

*“****Many women*** *(9/13) stated that they would have given up smoking if they had been given proof that it was dangerous, that the baby would be harmed:”^5^*

Every sentence referring to the population in which a quantifier is used (directly or indirectly referring to the population) should be included.

Condition 5b: Relationship Quantifier (RQ)

The author suggests something about the strength of the relationship by mentioning an ordering of factors or giving a weight to the factors using vague quantifiers.

*“Smoking was a familiar and* ***necessary tool*** *to cope.”^3^*

We have listed all identified relationship quantifiers in Table 2 (first column). The quantifiers in the text fragments can also refer to the uncertainty in which a statement about the relationship is made. Every sentence referring to the relationship in which a quantifier is used (directly or indirectly referring to the relationship) can be included in RQ.

*Ranking*

This section of the manual describes the procedure with regard to the ranking of the text fragments. Population and relation quantifiers are ranked separately. We show how we have ranked all the vague quantifiers present in the dataset.

Initially, four coders ranked the quantifiers separately (depicted in the first column of Table 2 and 3) and discussed them until ranking was agreed upon. We then clustered the ranks on a seven-, five- and three-point scale. The values are ranked with numbers in ascending order from very strong (highest rank 1) to very weak (lowest rank; 12, 7, 5, and 3 for the columns, respectively).

*Table 2*

Rankings of vague quantifiers PQ

| Vague quantifier population | Strength based on initial ranking | Strength based on seven-point scale | Strength based on five-point scale | Strength based on three-point scale |
| --- | --- | --- | --- | --- |
| Almost all | 1 | 1 | 1 | 1 |
| Nearly all | 1 | 1 | 1 | 1 |
| High percentage | 2 | 2 | 2 | 1 |
| Overwhelmingly | 3 | 2 | 2 | 1 |
| Prominent | 3 | 2 | 2 | 1 |
| Most frequently | 4 | 3 | 2 | 1 |
| Most | 4 | 3 | 2 | 1 |
| Majority | 4 | 3 | 2 | 1 |
| Often | 5 | 3 | 2 | 2 |
| Frequently | 6 | 4 | 3 | 2 |
| Generally | 7 | 4 | 3 | 2 |
| Common | 7 | 4 | 3 | 2 |
| Typically | 7 | 4 | 3 | 2 |
| Many | 8 | 5 | 4 | 2 |
| More likely | 9 | 6 | 4 | 3 |
| Several | 9 | 6 | 4 | 3 |
| Other | 10 | 6 | 5 | 3 |
| Sometimes | 11 | 7 | 5 | 3 |
| At times | 11 | 7 | 5 | 3 |
| Sometimes | 11 | 7 | 5 | 3 |
| A few | 12 | 7 | 5 | 3 |

*Table 3*

Rankings of vague quantifiers RQ

| Vague quantifier relation | Strength based on initial ranking | Strength based on seven-point scale | Strength based on five-point scale | Strength based on three-point scale |
| --- | --- | --- | --- | --- |
| Core/Central category | 1 | 1 | 1 | 1 |
| Main | 2 | 1 | 1 | 1 |
| Key | 2 | 1 | 1 | 1 |
| Major theme | 3 | 1 | 1 | 1 |
| Inextricably linked to | 4 | 2 | 1 | 1 |
| Essential | 4 | 2 | 1 | 1 |
| Necessary | 4 | 2 | 1 | 1 |
| Acute(ly) | 5 | 2 | 2 | 1 |
| Formidable | 6 | 3 | 2 | 1 |
| Powerful | 6 | 3 | 2 | 1 |
| Prominent | 6 | 3 | 2 | 1 |
| Very important | 7 | 3 | 2 | 2 |
| Great influence | 7 | 3 | 2 | 2 |
| Strong | 8 | 4 | 2 | 2 |
| Significant | 9 | 4 | 3 | 2 |
| Important | 9 | 4 | 3 | 2 |
| General theme | 10 | 5 | 3 | 2 |
| Common theme | 10 | 5 | 3 | 2 |
| Pervasive | 10 | 5 | 3 | 2 |
| Recurrent | 10 | 5 | 3 | 2 |
| Pattern | 10 | 5 | 3 | 2 |
| Considerable | 11 | 5 | 3 | 2 |
| Particular | 12 | 6 | 4 | 3 |
| Stressed | 12 | 6 | 4 | 3 |
| Highlighted | 12 | 6 | 4 | 3 |
| Especially | 12 | 6 | 4 | 3 |
| Emphasized | 12 | 6 | 4 | 3 |
| Magnified | 12 | 6 | 4 | 3 |
| Further reinforced | 12 | 6 | 4 | 3 |
| Likely | 13 | 7 | 5 | 3 |
| Other reasons | 14 | 7 | 5 | 3 |
| Minor importance | 15 | 7 | 5 | 3 |
| Marginal | 16 | 7 | 5 | 3 |

*Exception 1: Literature references*

Quotations that refer to literature are excluded for coding. In the discussion or results sections, authors might pose statements referring to other literature. Whenever a text fragment containing the relationship and the quantifier refers to a literature statement, this statement is excluded from the analysis as it is not part of the findings of the included study. An example:

*“The women in this study thought that their partners and family/friends would be supportive of them quitting smoking, although it was clear that this support did not extend to these significant others stopping smoking. Emotional support from partners and close family/friends has been shown in the past not to be associated with smoking cessation,* ***whereas actual smoking behaviour change from these significant others was independently associated with quitting smoking (Appleton and Pharoah, 1998).****”^11^*

In the case that a finding of the study under review is connected to literature reference (for example: the findings of this study *underline* the findings of (ref) that women often quit smoking (…)), it should be included in the codings, as the study itself actually established this relationship.

*Exception 2: Repetition*

There are instances in which the author repeats the same finding throughout a paragraph or the whole paper. The criteria for excluding a fragment from the analysis based on repetition are 1) the quantifier is exactly the same 2) words used to indicate the relationship are exactly the same.

*“Abstract*

*Most women who smoked took active steps to reduce the risks. Actions were* ***frequently accompanied by beliefs that rationalized moderate levels of smoking****.”^12^*

*“Results*

*In addition, active steps (e.g., cutting back, changing brands, and quitting)* ***were frequently***

***accompanied by beliefs*** *about the risk of one’s own smoking habits that, together with these actions, helped to* ***rationalize moderate levels of smoking.****”^12^*

For other cases in which the author elaborates on a particular earlier mentioned finding, it might be less straightforward whether a finding should be coded twice. It could be that the author clarifies or exemplifies a particular situation. The following quotation illustrates such a situation:

*“****Many participants*** *talked about* ***being reassured by others*** *– friends, family and health professionals – that their* ***continued smoking*** *in pregnancy was* ***not causing much harm****. This appeared to make it easier to psychologically resolve smoking related dissonance.* ***Most women*** *who continued to smoke, but had cut down, indicated that they had received a positive response from a health professional (usually their midwife) in response to them reporting this reduction. This response was taken by some as guidance as to what was* ***acceptable to minimise smoking related risks****:”^13^*

One could argue that in the second part of the text fragment, starting with “most women”, a new fragment starts. However, the first sentence is a general statement about a relationship which is then further specified in the next sentence; which means, referring to the same finding. In cases where the author states a finding and then elaborates on that for clarification, the finding is not coded twice. Instead, the coder can choose to include the clarification into the coded fragment. In some instances where this happens, signaling words like “for example”, “more specifically”, or “in particular” are used.

This general repetition rule also holds for mixed methods studies in which results from the quantitative and qualitative data are being discussed in combination after being discussed separately.

**References**

*Studies marked with an asterisk indicate studies included in the review.

1. Boeije, H, Van Wesel, F, Slagt, M. Guidance for deciding upon use of primary mixed methods studies in research synthesis: lessons learned in childhood trauma.  *Qual Quant.* 2014; 48(2): 1075-1088.

2. *Haslam, C, Draper, E. A qualitative study of smoking during pregnancy. *Psychol Health Med.* 2001; 6(1): 95-99.

3. *Tod A. Barriers to smoking cessation in pregnancy: A qualitative study. *Br. J. Community Nurs.* 2003; 8(2): 56–64.

4. *Edwards N, Sims‐Jones, N. Smoking and smoking relapse during pregnancy and postpartum: results of a qualitative study. *Birth*. 1998; 25(2): 94-100.

5. Arborelius E, Nyberg K. How should midwives discuss smoking behaviour in pregnancy with women of low educational attainment? *Midwifery*. 1997; 13(4): 210–215.

6. *Abrahamsson, A, Springett, J, Karlsson L, Ottosson J. Making sense of the challenge of smoking cessation during pregnancy: a phenomenographic approach. *Health Educ Res.* 2005*;* 20(3): 367-378.

7. Sechrest, L, Sidani, S. Quantitative and qualitative methods: Is There an Alternative? *Eval Program Plann*. 1995; 18(1): 77-87.

8. Sandelowski M. Real qualitative researchers do not count: The use of numbers in qualitative research. *Res Nurs Health.* 2001; 24(3): 230-240.

9. Chang, Y, Voils, CI, Sandelowski, M, Hasselblad, V, Crandell, JL. Transforming verbal counts in reports of qualitative descriptive studies into numbers. *West J Nurs Res*. 2009; 31(7): 837-852.

10. Onwuegbuzie AJ. Effect sizes in qualitative research: A prolegomenon. *Qual Quant.* 2003; 37(4): 393-209

11. *Thompson KA, Parahoo KP, McCurry N, O’Doherty E, Doherty AM. Women’s perceptions of support from partners, family members and close friends for smoking cessation during pregnancy: combining quantitative and qualitative findings. *Health Educ Res.* 2004; 19(1): 29–39.

12. *Dunn CL, Pirie PL, Lando HA. Attitudes and perceptions related to smoking among pregnant and postpartum women in a low-income, multiethnic setting. *Am J Health Promot*. 1998; 12(4): 267–274.

13. *Naughton F, Eborall H, Sutton S. Dissonance and disengagement in pregnant smokers: a qualitative study. *J Smok Cessat.* 2013; 8(1): 24–32.
